# Experimental Study of Thermal Behavior of Insulation Material Rigid Polyurethane in Parallel, Symmetric, and Adjacent Building Façade Constructions

**DOI:** 10.3390/polym10101104

**Published:** 2018-10-06

**Authors:** Xin Ma, Ran Tu, Xudong Cheng, Shuguang Zhu, Jinwei Ma, Tingyong Fang

**Affiliations:** 1 College of Environment and Energy Engineering, Anhui Jianzhu University, Hefei 230022, China; maxin@mail.ustc.edu.cn (X.M.); turangreat@sina.com (S.Z.); zhou-xj@hotmail.com (J.M.); 2 College of Mechanical Engineering and Automation, Huaqiao University, Xiamen 361021, China; 3 State Key Laboratory of Fire Science, University of Science and Technology of China, Hefei 230026, China; antigy@mail.ustc.edu.cn

**Keywords:** adjacent façade, PUR, energy conservation, heat transfer, burning characteristic

## Abstract

Both experimental and theoretical methods were proposed to assess the effects of adjacent, parallel, and symmetric exterior wall structures on the combustion and flame spreading characteristics of rigid polyurethane (PUR) foam insulation. During the combustion of PUR specimens, the flame leading edge was found to transfer from a unique inverted ‘W’ shape to an inverted ‘V’ during flame propagation. This phenomenon is attributed to edge effects related to boundary layer theory. The effects of the adjacent façade angle on flame spreading rate and flame height were shown to be nonlinear, as a result of the combined influences of heat transfer, radiation angle, and the chimney restriction effects. A critical angle around 90 degree with maximum thermal hazards outwards by parallel fire was observed and consistent with the mass loss rate and flame height tendencies. For narrow spacing configurations or angles (e.g., 60 and 90 degrees), phenomenological two-pass processing in conjunction showed that increased preheating lengths were associated with enhanced heat transfer. The results of this study have implications concerning the design of safe façade structures for high-rise buildings, and provide a better understanding of downward flame spreading over PUR.

## 1. Introduction

**Figure 1:**
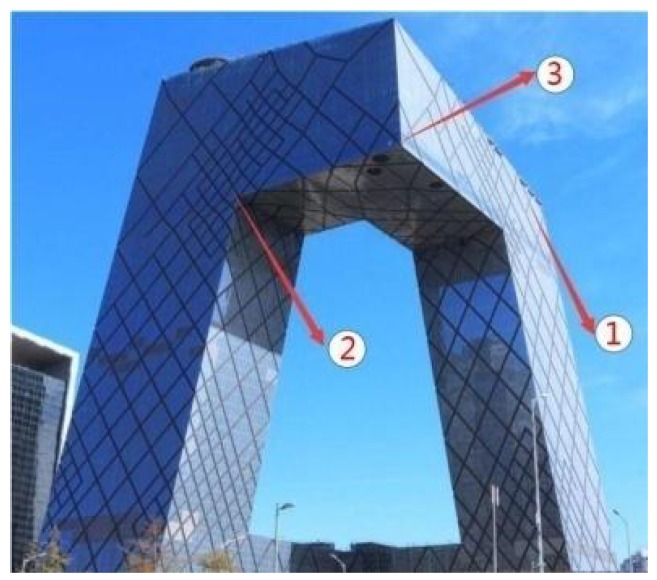
The complex coupled adjacent wall structure of the Beijing Television Cultural Center, including vertical external, vertical internal, and horizontal external corner structures.

Modern designs for the construction of building exterior façades tend to be complex as a result of requirements related to lighting and aesthetics. Different kinds of adjacent wall coupling configurations are used, such as that employed in the Beijing Television Cultural Center with varied inner or outer angles shown in Figure 1. One important aspect of building façade structure design is fire safety. As an example, the flame spreading characteristics associated with vertical adjacent façades can complicate fire rescue operations in high-rise buildings, because certain configurations significantly affect the air entrainment and flow field around the exterior façade. Unique flame spreading behavior may appear if a fire occurs on a building façade associated with an adjacent wall.

There has been considerable research regarding the safety and risk analysis of energy conservation materials, especially related to exterior thermal insulation materials, with the aim of developing fundamental guidelines for the design, construction, and operation of buildings. 

Interest in energy conservation has continually increased over the last two decades [1,2]. As a result, it has been determined that improving building insulation based on rigid polyurethane (PUR) can substantially reduce energy consumption and therefore lower carbon emissions, which in turn may serve to reduce climate change. PUR is widely employed because the materials serve as heat shields and can also be visually attractive, but unfortunately, it could be easily ignited and exhibit extremely fast flame spreading rates during fire hazards. Previous research has demonstrated that the combustion behavior of PUR foam results in challenges when designing fire protection systems for buildings. In fact, there have been several large fires partly attributable to the presence of PUR as a thermal insulation material on the exterior walls of a building, such as the London Grenfell Tower fire in 2017 [3,4], which caused at least 80 deaths and over 70 injuries. Previous studies have mainly considered single fire sources rather than multiple flame scenarios, and little work was focused on flame spreading in association with the interaction of multiple flames. In an actual fire, the melting and dripping behavior of combustible materials, as well as the presence of winds, can cause high temperature flammable substances to detach from the original flame location to start new fires at other sites. In the case that adjacent building façades are sufficiently close, multiple fires can spread in parallel, increasing the fire spreading rate and complicating rescue efforts. Thus, the unique parallel flame spreading phenomenon associated with different adjacent façade configurations should be studied for better understanding of the downward flame spreading mechanism and to improve fire prevention and control.

Previous theoretical and experimental studies have determined the effects of various building façade configurations on solid fuel flame spreading behavior. de Ris [5] developed the early significant analytical model for flame spread in a forced convection environment with an exact solution in the thick limit and an approximate solution. Bhattacharjee et al. [6,7] extended the de Ris’s theory to downward spreading configuration by proposing an empirically determined equivalent buoyant convection velocity. The predictions which retain the functional form of the de Ris formula, are shown to accord well with computational and experimental results. We [8,9] studied flame spreading during the combustion of PUR foam at low pressures and at various façade inclinations. Empirical relationships were deduced between pressure and the average flame spreading rate at different façade angles, which can be summarized as $V_{a}\propto p^{n},0.65<n<0.89$ (where $V_{a}$ is the average flame spread rate and p is the air pressure). Umberto [10] reported the fire characteristics including mass loss rate and temperature variation of fiber reinforced polymer slabs. Quintiere [11] studied the effect of inclination angle on flame spreading over thin substrates and determined that the relationship between the total heat flux from the flame and the sample incline is ${\dot{q}}_{f}^{\prime \prime }=C_{q,L}BL{\left(\sin\vartheta L\right)}^{2/5}x_{p}^{1/5}$ (where ${\dot{q}}_{f}^{\prime \prime }$ is the flame heat flux, ϑ is the inclination angle, and $x_{p}$ is the pyrolysis length ). Kashiwagi [12] investigated the effects of sample orientation during inclined downward flame spreading over various solid fuels using both experimental and theoretical methods. Experimental results showed a linear relationship, $v_{f,opp}={\left(\sin\vartheta \right)}^{1/3}$ (where $v_{f,opp}$ is the opposed flame spread rate). Similar results were also obtained by Zhou [13] and Sibulkin [14]. Shi [15] used mathematical model to describe the pyrolysis and combustion processed of different polymers. An [16] investigated the effects of a parallel curtain wall on the downward flame spreading characteristics of insulation materials on a building façade, using a single linear ignition source. The average flame height and maximum flame temperature were found to initially become lower and then increase with increasing spacing due to the coupled chimney and restriction effects induced by the curtain wall. The theoretical equations related to diffusion flame spreading over solid combustible surface were investigated by Huan [17] and Kurosaki [18] based on the introduction of multiple flames, and different conclusions were obtained. Huan [17] conducted experiments to elucidate the flame spreading mechanism over two parallel slabs in either vertical or horizontal direction with varying separation distances. It demonstrated that flame spreading is determined by a variable convection coefficient and that the mass loss and flame spreading rates both initially increase and then decrease with increasing separation spacing. Kurosaki [18] investigated the steady, two-dimensional vertical downward spreading of flame along two parallel paper sheets. The experimental and theoretical results showed that convective heat transfer is dominant in the case of a narrow space between the burning paper sheets. In contrast, radiation from the opposite flame and embers plays an important role in controlling the flame spreading rate in the case of wider spacing. In general, studies of the effects of adjacent façades or multiple fire sources on flame spreading characteristics over combustible surface have been limited. A deeper understanding of the combustion behavior of façade materials with adjacent materials at various angles is vital to the fire safety design of buildings incorporating energy saving insulation systems.

In the present work, comparative bench-scale experiments were conducted to investigate the burning behavior of PUR foam board in conjunction with downward flame spreading under various conditions. PUR foam boards were ignited by a propane flame as a linear fire source to generate a parallel, symmetric flame starting at the top of each specimen. This work examined the effects of various adjacent façade constructions on PUR foam board combustion rates, vertical flame heights, and flame spreading characteristics. An analysis of the associated heat transfer mechanism was also performed. 

## 2. Materials and Methods

**Figure 2:**
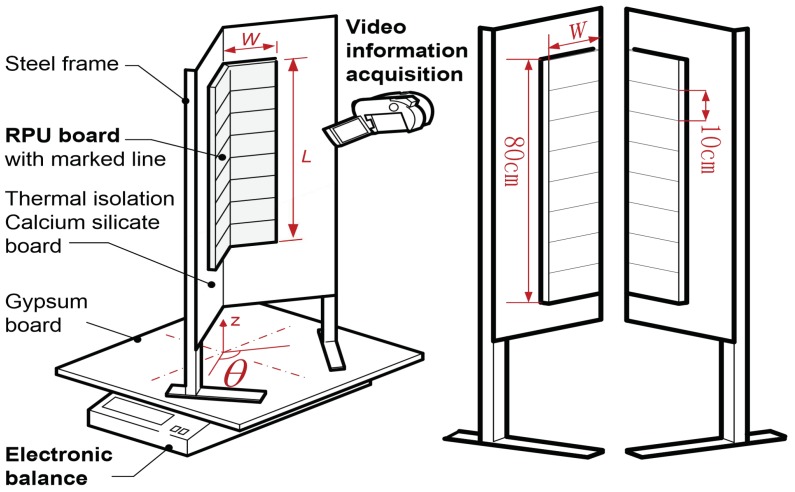
Experimental setup used to study polyurethane (PUR) board combustion behavior in conjunction with various adjacent façade constructions.

The experimental apparatus primarily consisted of an electric balance, a PUR board holder, sensors and a measurement system as shown in Figure 2. Two PUR foam boards with the same size (2 cm thick, 80 cm long and 10 cm wide) were mounted on the holder, which in turn stood on an insulating gypsum board. Both PUR boards were ignited at its upper part to initiate unrestricted downward flame spreading. The angle between the two boards could be adjusted, and five adjacent façade incline angles (ϑ = 60°, 90°, 120°, 150°, or 180°) were employed during these trials. The properties of the PUR foam board selected for these experiments are listed in Table 1.

The PUR board holder with gypsum board was situated on an electronic balance with an accuracy of 0.01 g to allow monitoring fuel mass variation. An ethanol-soaked wick held in an iron niche was used to achieve linear ignition of the foam. A TS-30 radiation flux meter was located 1.5 m in front of the adjacent façade configuration with the same height as the middle of the fuel board, measuring the thermal radiation of parallel fire outwards to the surrounding environment. Two high definition digital cameras were employed to record the flame spreading and variations in the leading edge position in real time from side view and top view of the boards. Because the thermal conductivity of the gypsum board is minimal, heat loss from the flame to the vertical façade via conduction was negligible and therefore had little effect on flame development.

All experiments were conducted at a constant initial air temperature and humidity (22.0 ± 2.0 °C, 55% ± 4% relative humidity). All temperature and fuel mass data were recorded at a frequency of 1 Hz. Repetition of the experiments was performed until results were confirmed to be reproducible.

## 3. Results and Discussion

### 3.1. Flame morphology and Spreading Behavior

**Figure 3:**
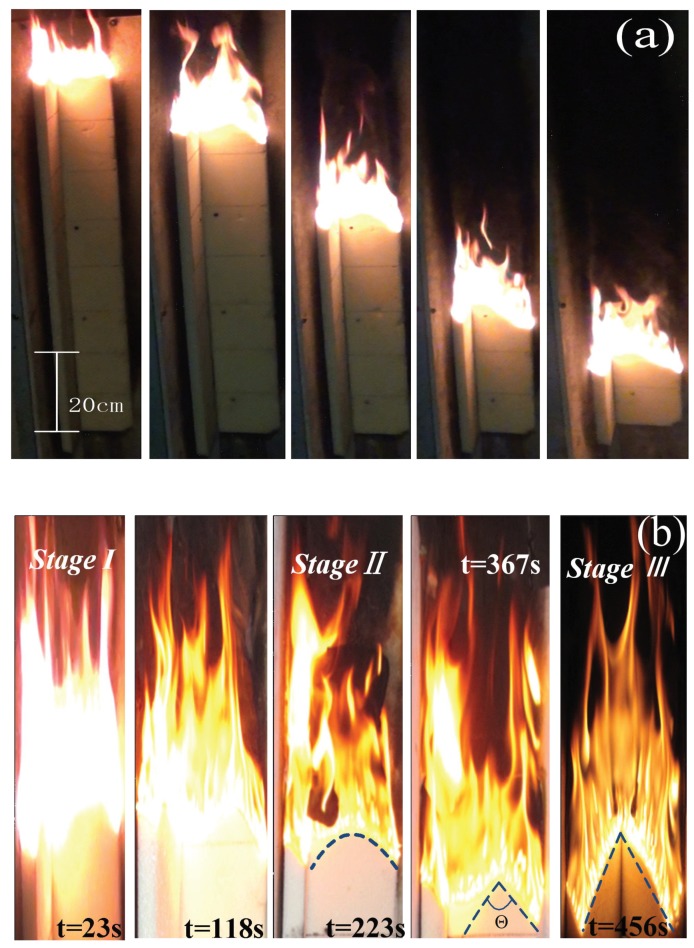
(**a**) Sequential images of the downward burning behavior and (**b**) variations in the flame leading front of adjacent PUR board with an adjacent façade angle of 90°.

**Figure 4:**
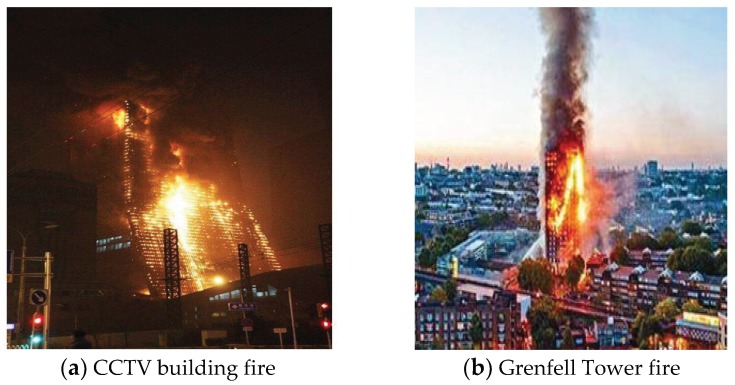
Images of the CCTV building and Grenfell tower fires.

**Figure 5:**
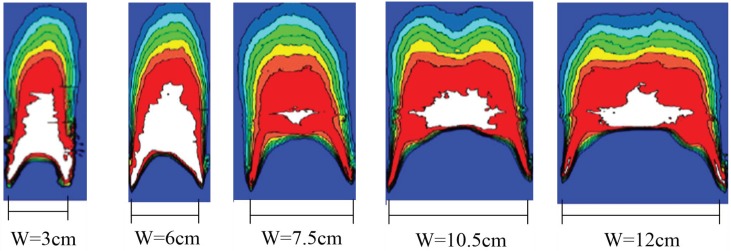
Variations in the morphology of the flame leading edge with width W using PMMA specimens, as published by Gong [19].

In the downward flame spreading process, the controlling mechanism is based on heat and mass transfer to the unburned area. The unique dynamic, parallel, and symmetric combustion scenario in this work resulted in morphological variations of the pyrolysis leading edge (or flame front) of the PUR foam as shown in Figure 3. Images of typical sequential downward flame spreading are shown in Figure 3a. These variations in the leading edge can be divided further into three stages (Figure 3b), during which a distinctive inverted ‘W’ shape changes to an inverted ‘V’ shape. Initially, after the flame has travelled for about 10 cm, the flame spreading reaches to an approximate steady state, and the flame pyrolysis front show essentially one-dimensional linear flame spreading (Stage 1 in Figure 3b). The flame front at the board edges spreads faster than at the center, so that the flame front becomes more irregular, with an inverted ‘V’ shape for each board (Stage 2 in Figure 3b). Eventually, an inverted parallel symmetric ‘W’ shaped flame front composed of two inverted ‘V’ shapes is observed, indicating two-dimensional flame spreading. Finally, the lateral flame front spreading becomes more rapid, leading to a ‘slash’ shape (Stage 3 in Figure 3b), and an inverted ‘V’ shape composed of two slash-shaped leading fronts emerges. The inverted ‘V’ shape appearing in our experiments is similar to the flame morphologies observed in the CCTV and Grenfell tower accidents (Figure 4). Prior work has also demonstrated the formation of an inverted ‘V’ shape leading edge with solid fuel flame spreading, although not in all cases. In the case of wide boards, the leading edge has been found to exhibit an inverted ‘U’ morphology rather than a ‘V’ shape, indicating an edge effect caused by air entrainment from both sides, as reported by Gong using polymethyl methacrylate (PMMA, Figure 5) [19]. These variations in the flame leading edge are discussed in detail in Section 3.3.

### 3.2. Overall Comparison of Burning Rates

**Figure 6:**
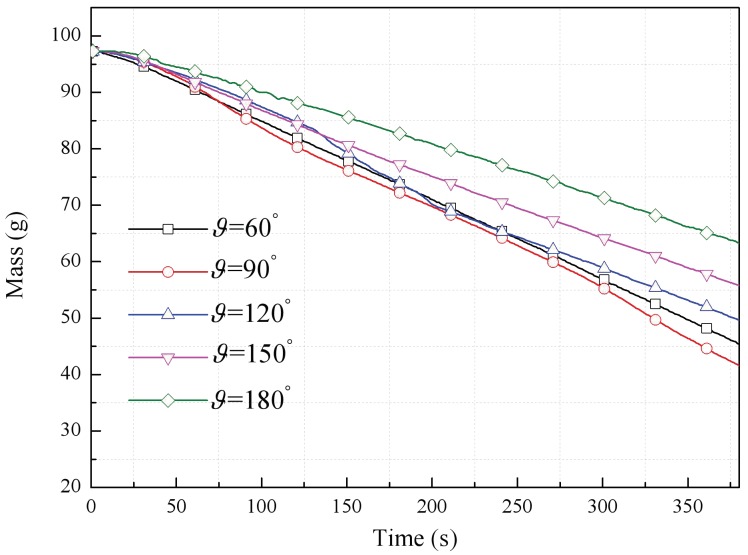
PUR mass losses as functions of time at various angles.

Figure 6 presents a comparison of mass loss data acquired during the relatively steady burning stage in conjunction with different adjacent façade configurations. The extent of complete combustion, η, can be used to investigate the effects of a parallel symmetric flame and entrainment of the fire plume with changes in the adjacent angle. This term is defined as $\eta =\frac{m_{r}}{m_{i}}$, where $m_{i}$ is the initial mass of the PUR foam board, and $m_{r}$ is the mass remained after extinguished or the mass of the char when the flame spreading process completed.

Table 2 summarizes the η values obtained for specific angles. The relationship between the PUR burning rate and the adjacent façade angle is seen to be nonlinear, i.e., η first decreases with increases in the angle and then increases. This phenomenon is thought to result from a coupling effect based on heat feedback from the opposite flame and air entrainment due to the chimney effect. Both are modified by changing the adjacent angle, as discussed below.

The PUR burning rate for each angle is largely dependent on heat transfer from the flame. Comparing the data for $\vartheta =60^{{}^{\circ}}$ (η=34.10%) and $\vartheta =90^{{}^{\circ}}$ (η=31.78%), the two parallel flames evidently affect one another to different extents as the angle is changed by both radiative and convective heat transfer. When the angle is decreased, radiation heat feedback is strengthened. However, increased entrainment of cold air into the flame plume in the gap between the façades (due to the chimney effect) with decreases in the angle could cool the combustion zone, resulting in a decreased burning rate.

With the enlarged angle, such as $\vartheta =120^{{}^{\circ}}$ (η=33.20%), it further decreased the heat transfer from the opposite flame even though a sufficient air supply was available, leading to weakened combustion compared with a more narrow façade construction. Above a specific angle, the mutual reinforcement effect was greatly decreased and become negligible, reducing the combustion intensity, e.g., occurred at 150° (η=40.48%) and 180° (η=51.52%).

**Figure 7:**
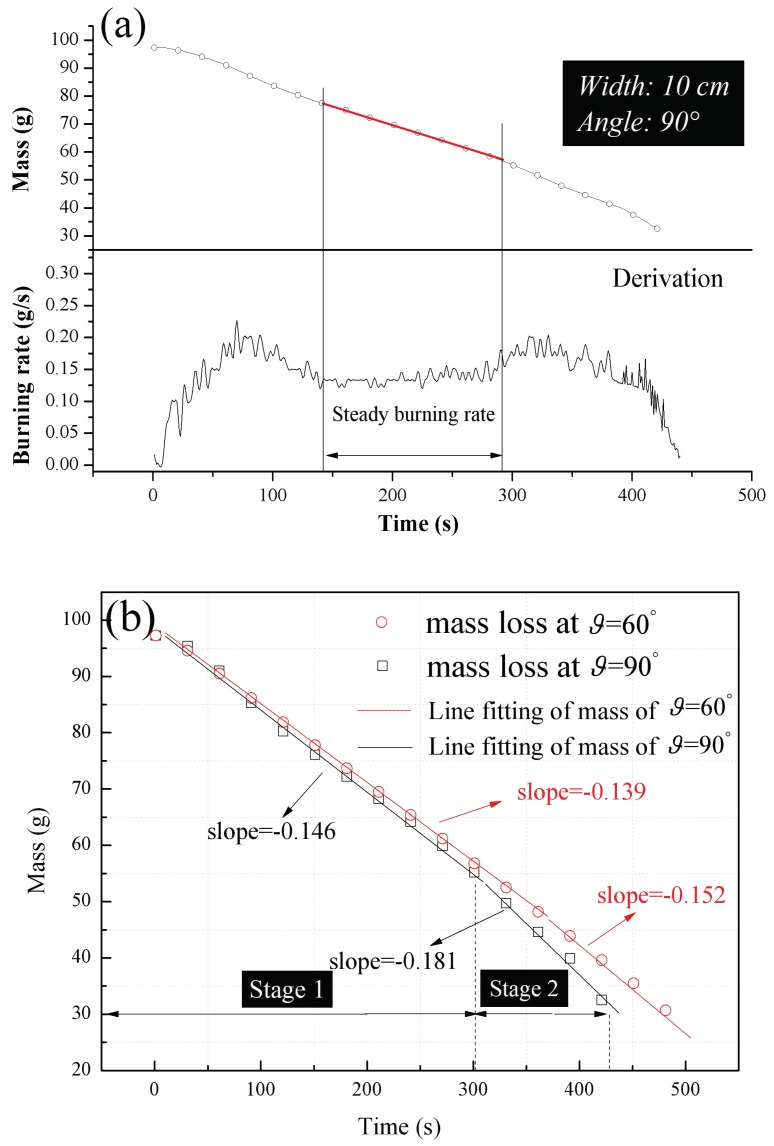
(**a**) The mass loss and burning rate, $m^{\prime \prime }$ (calculated as the derivative of the mass loss) and (**b**) the phenomenological two-pass processing of data acquired in the later flame spreading stage, both as functions of time.

Throughout this work, the burning rates for comparison were selected from the relatively steady flame spreading period, as obtained using the linear fitting method depicted in Figure 7a (based on data acquired at a 90° adjacent façade angle). During this stage of combustion, a plot of the pyrolysis front position against time is nearly linear at first. However, a sudden, unexpected increase in the mass loss rate was observed with phenomenological two-pass processing, dividing the data into two stages with different slopes. This increase indicates an acceleration of the downward flame spreading during the later period of combustion over a narrow range of angles ($\vartheta =60^{{}^{\circ}}$ and $\vartheta =90^{{}^{\circ}}$), as shown in Figure 7b. In such cases, the data plot is closer to parabolic than linear over a sufficiently long time scale. Taking $\vartheta =90^{{}^{\circ}}$ as an example, if flame spreading is monitored over approximately 300 s, the mass loss rate is constant at 0.146 g/s during the initial stage but later abruptly increases to 0.181 g/s. This effect is attributed to preheating of the unburned region of the PUR via heat transfer from the flame, leading to a widened preheating zone and less heat is required for pyrolysis of the PUR by preheated. Thus, the flame front reaches the pyrolysis temperature more quickly during the later period, which in turn accelerates the flame spreading.

### 3.3. Flame Height and Flame Front Variations

**Figure 8:**
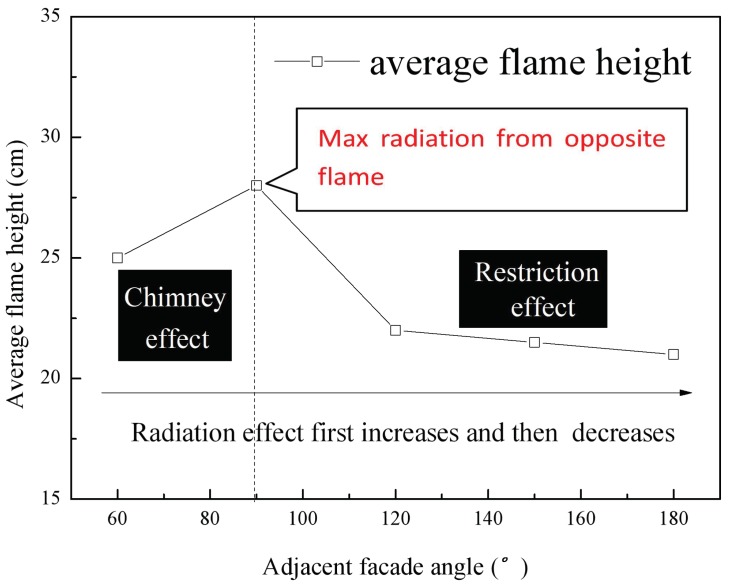
Flame height as a function of the adjacent façade angle.

**Figure 9:**
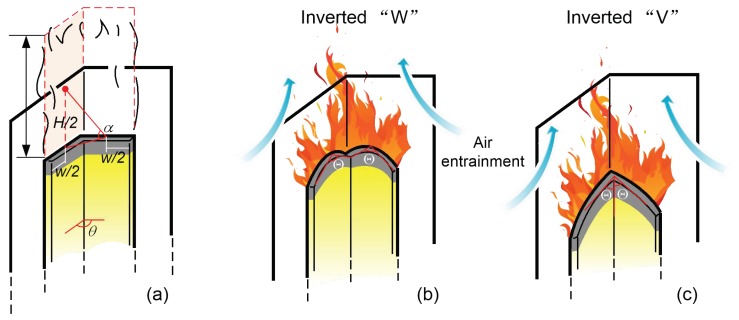
Schematic showing variations in leading front flame spreading induced by the edge effect.

The average flame heights at various façade angles are plotted in Figure 8, and this plot shows that the flame height first increased and then decreased with enlarged angle. A critical angle $\vartheta_{c}$ is noticed at approximately $\vartheta =90^{{}^{\circ}}$ in this plot, similar to the trend exhibited by the burning rate vs. angle, which is not surprising because the burning rate is the key parameter determining the flame height, spreading velocity and other factors according to classical fire dynamic theory. The variation in the average flame height is significant at $\vartheta <\vartheta_{c}$ but little change is seen above this value. These data can be explained based on radiation, chimney, and restriction effects. Considering that the radiation effect is the most important thermal feedback parameter in this situation, it changes not linearly with the angle (see Figure 9). The radiation heat feedback $q_{r}$ to the preheating zone for each board consists of that from the sample itself $q_{{}_{rs}}^{\prime \prime }$, and that from the adjacent façade $q_{ra}$. However, $q_{{}_{rs}}^{\prime \prime }$ is negligible because the view factor $F_{s}$ is small enough. Therefore, we can write

(1) $$q_{{}_{r}}^{\prime \prime }=q_{rs}^{\prime \prime }+q_{{}_{ra}}^{\prime \prime }\;$$

(2) $$q_{rs}^{\prime \prime }=\varepsilon \sigma (T_{{}_{S}}^{4}-T_{a}^{4})F_{s}\;$$

(3) $$q_{\prime \prime }^{ra}∼\frac{Q_{ra}\cos\alpha }{4\pi R^{2}}\sin\vartheta .$$

The approximately value of $q_{{}_{ra}}^{\prime \prime }$ can be obtained from Equation (3), where $Q_{ra}$ is the total heat flux transfer to the adjacent façade and R is the length from fire center to the burning zone of opposite adjacent façade. α is the approximate radiation angle between the opposite flame and the board pyrolysis zone. Based on fire dynamics, the flame radiation can be assumed to be emitted in a spherical pattern from a geometrical center. Due to the relatively small PUR board width, the radiative heat flux at this point can be considered to be equal to the average flux received from the opposite flame. According to Equation (1), with decreases in ϑ, the flame radiation output increases. If the adjacent angle is $\vartheta {=180}^{{}^{\circ}}$, the façades are parallel to one another and $q_{{}_{ra}}^{\prime \prime }$ will be low. In contrast, at $\vartheta {=90}^{{}^{\circ}}$, the heat flux between adjacent façades will be the highest and the mutual fire sources will impact each other to the maximum extent.

As the adjacent façade angle increases, the chimney effect decreases and the flame height drops. Thus, when $\vartheta =\vartheta_{c}$ at a value of approximately 90°, the maximum burning rate and flame height are obtained. In the case that $\vartheta >\vartheta_{c}$, the radiative and chimney effects are both greatly decreased, while the restriction effect is also weakened such that the flame height slightly decreases. Therefore, the flame height first increases and then drops with increases in the adjacent façade angle. 

The flame front or pyrolysis edge are determined by several factors, including flame height, air entrainment, and the time over which the fire has developed. As aforementioned, the leading edge changed from an inverted ‘W’ shape to an inverted ‘V’ shape. This phenomenon can be interpreted based on Gollner’s [20] boundary layer theory which has its basis in the work of Chilton and Colburn [21] and Silver [22], whose extension to the Reynolds’ analogy established a relationship between mass, momentum, and heat transfer in a boundary layer over the fuel surface. The associated equation is

(4) $$\frac{\tau_{s}}{u_{\infty }v^{2/3}}=\frac{{{\dot{m}}_{t}}^{\prime \prime }}{D^{2/3}\ln(1+B)}\;$$

where the air shear stress, $\tau_{s}$, is the viscosity coefficient multiplied by the derivative of velocity. This term can be written as

(5) $$\tau_{s}=\mu (\frac{\partial u}{\partial y}+\frac{\partial v}{\partial x}).$$

In addition, $\mu_{\infty }$ is the free-stream velocity, v is the kinematic viscosity or momentum diffusivity, ${\dot{m}}_{t}{}^{\prime \prime }$ is the mass transfer caused by shear flow, D is the species diffusivity, and B is the Spalding number is the mass transfer coefficient. This value is determined using the equation

(6) $$B=(\Delta H_{c}fY_{\infty }+C_{pg}\Delta T)/(L+C_{ps}\Delta T_{s}).$$

Hence, the mass transfer rate is positively correlated with $\tau_{s}$**,** the effect of which is obvious during solid combustion due to an additional induced effect. The inverted ‘W’ flame front is attributed to three effects. First, the flame size will be larger at the specimen sides than in the center of the board. Second, shear entrainment from the lateral sides will accelerate the flame spreading velocity in these locations. At last, as the flame height and temperature reach their maximum values at the center of the adjacent board, the burning rate will be increased. In the later stage of combustion, due to the enhanced burning at the board sides by the effect of $\tau_{s}$, the spreading velocity at the sides will become significantly faster so as to form the inverted ‘V’ shape.

The angle Θ of the inverted ‘V’ shape was found to decrease as flame spreading progressed. This effect can be expressed by the equation simplified from Gong’s research [19]

(7) $$\Theta \sim \arcsin(\frac{q_{\delta }^{\prime \prime }+q_{p}^{\prime \prime }}{\rho V_{f}[c(T_{p}-T_{\infty })]})\;$$

where $q_{\delta }^{\prime \prime }$ is the radiative heat feedback in the preheating zone, $q_{p}^{\prime \prime }$ is the thermal feedback in the combustion zone, and $V_{f}$ is the flame spreading rate, defined as the propagation speed of the flame front along the sample surface. The smallest value of Θ was observed at $\vartheta {=90}^{{}^{\circ}}$ also associated with tangential entrainment effect, plus parallel fire thermal feedback is the largest resulting in peak combustion rate. Meanwhile, the flame height and maximum entrainment strength were enhanced further, and positively feedback the combustion efficiency of board edges, finally resulting in a sharp pyrolysis front.

### 3.4. Flame Spreading Rates

**Figure 10:**
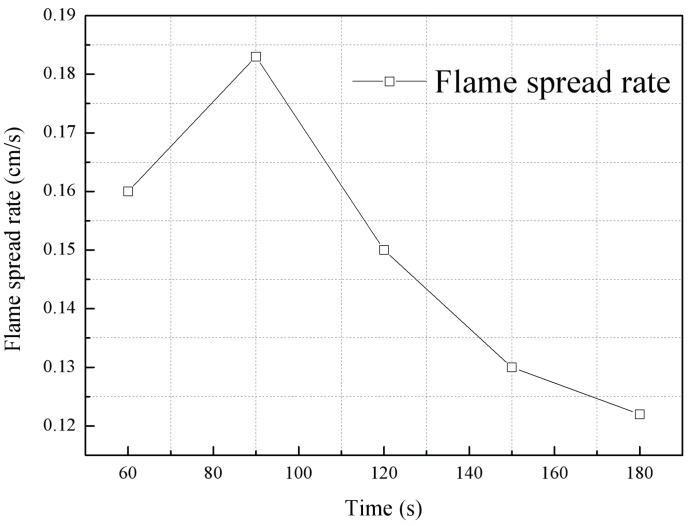
Average flame spreading rates during the stable flame spreading stage as a function of time.

The flame spreading rate is impacted by the disturbance associated with the ignition source in the early stage of combustion and the accelerated flame spreading during the later stages. Hence, the stable flame spreading stage was employed when determining the $V_{f}$ values, as shown in Figure 10. 

**Figure 11:**
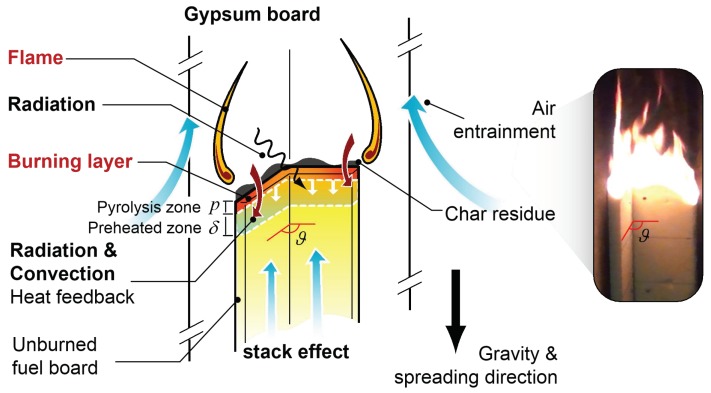
A diagram and photographic image showing downward, parallel, and symmetric flame spreading behavior.

Quintiere [11] proposed a simplified theory to predict the downward flame spreading rate over a thermally thick charring solid, based on the equation

(8) $$V_{f}=\frac{1}{\rho c\cdot wd(T_{p}-T_{\infty })}\int_{0}^{p+\delta }\left(q_{c}{}_{d}+q_{r}+q_{cv}\right)dx\;$$

where ρc is the density multiplied by the specific heat, wd is the fuel width multiplied by the thickness, p is the pyrolysis length, and δ is the preheating length. In the case of downward flame spreading with adjacent materials at various angles, the heat feedback is largely determined by the radiative heat flux $q_{r}$ (which could be negligible for single board flame spreading), convective heat flux $q_{cv}$ and conductive heat flux, $q_{cd}$. A diagram depicting downward parallel, symmetric flame spreading is presented in Figure 11. Also, de Ris [5] and Bhattacharjee et al. [6,7] proposed a formula to predict the downward flame spread rate of thermal thick solid, as shown in Equation (9).

(9) $$V_{f,thick}∼\frac{\lambda_{g}\rho_{g}c_{g}{\left(T_{f}-T_{v}\right)}^{2}}{\lambda_{s}\rho_{s}c_{s}{\left(T_{v}-T_{\infty }\right)}^{2}}\;$$

which is an empirical relationship only for single board condition, without considering more radiation interaction.

Anyhow, the convective heat flux can be expressed as

(10) $$q_{cv}=h_{c}\frac{\partial T}{\partial y}|{}_{y=0}.$$

As the adjacent angle or space between the two boards decreased, the stack effect will become prominent, such that upward air entrainment is strengthened. As a result of the cold air cooling effect, the convective heat transfer is reduced, although the effect of this heat transfer mechanism is minimal in the case of a narrow board. It is only when the sample width is extended that the weakening effect of the convective heat feedback will have a significant effect on flame spreading.

The value of $q_{cd}$ is approximately 0.03w/(m⋅k) for PUR foam, which affects the flame spreading behavior to a greater extent as the preheating length δ. Due to the increase in the radiation heat feedback when the surface temperature of the entire board is sufficiently high, the flame spreading characteristics of a single board and of two adjacent boards become quite different especially in the later period. The initial temperature of the unburned region rises significantly, and so the heat input required to achieve vaporization and ignition is reduced. This effect could increase the depth to which the conductive heat from the flame front penetrates, leading to a larger preheating zone.

**Figure 12:**
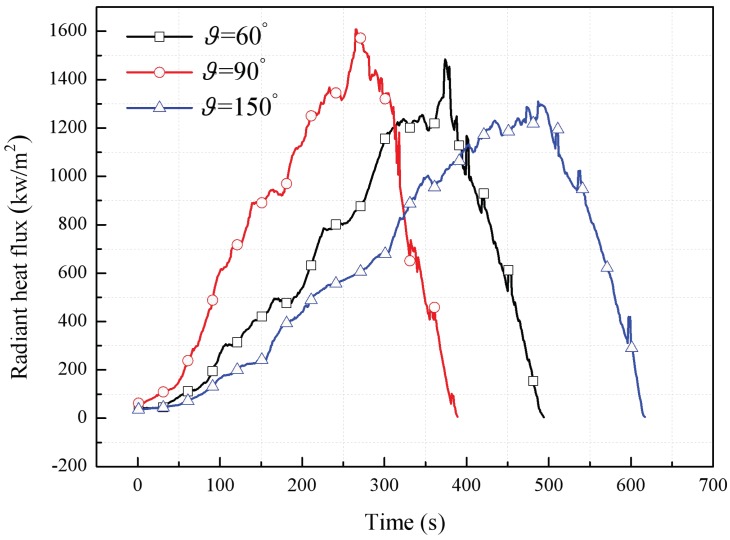
Radiant heat flux of parallel and symmetric flame with different angle constructions.

Because radiation heat feedback is stronger during downward flame spreading in the case of two parallel adjacent façades, this mechanism will be more important than convective and conductive heat transfer. Thus, the flame spreading rate will be largely determined by radiative feedback, which varies in a similar trend to the burning rate. In spite of internal radiant heat feedback, according to the measurement results by radiation flux meter, the radiation heat flux to external environment were compared as shown in Figure 12. It can be seen that the maximum value represents largest thermal hazard also appears at 90° condition, which is also consistent with the mass loss rate and flame height trends.

## 4. Conclusions

This work reported the two-dimensional vertical downward, parallel, and symmetric flame spreading characteristics of PUR foam. The correlations among the flame spreading velocity, mass loss rate, flame height, and adjacent façade structure effects were determined based on experimental data and theoretical relationships. The results could be helpful with respect to fire hazard assessment and safety design of adjacent building façades. The following conclusions can be made.

1. The parallel symmetric flame leading edge front was observed to change from one-dimensional to a unique morphology with an inverted ‘W’ shape, and finally exhibited an inverted ‘V’ shape. This occurred in conjunction with a narrow sample width due to the edge effect and the shear force that supplied additional heat feedback with enriched oxygen diffusion. The angle of the inverted ‘V’ shape was found to decrease as the flame spreading progressed.
2. As the adjacent façade angle decreased, the burning rate varied in a nonlinear manner. This is attributed to the competition between the negative and positive effects of the parallel adjacent façade configuration. The downward flame spreading over the PUR was essentially stable during the early stage of flame spreading while accelerated flame spreading was observed during the later period. Phenomenological two-pass processing of the mass loss data showed complex combustion behavior that could complicate fire rescue.
3. The average flame spreading rate and flame height both initially increased and then decreased with increases in the adjacent angle, similar to the trend displayed by the burning rate data. A critical angle of approximately $\vartheta {=90}^{{}^{\circ}}$ was identified, due to the combined chimney and restriction effects induced by changes in the adjacent façade configuration. At smaller angles ($\vartheta {=60}^{{}^{\circ}}$ to $\vartheta {=90}^{{}^{\circ}}$), the radiative heat transfer increased as a result of increases in the heat transfer from the opposite flame and the weakened chimney effect. At larger angles, the radiative heat transfer from the opposite flame and ember was gradually decreased, resulting in a lower flame spreading rate.

## Figures and Tables

**Table 1:** Properties of the PUR foam used for tests.

Tested Material	Density (kg/m^3^)	Heat Capacity (J/kg·K)	Thermal Conductivity coefficient (W/m·K)	Pyrolysis Temperature (K)	Heat of Combustion (MJ/kg)
PUR	60	1300	0.03	470	27

**Table 2:** Fuel combustion percentages at various adjacent façade angles.

Percentage (%)	60°	90°	120°	150°	180°
η	34.10%	31.78%	33.20%	40.48%	51.52%
